# An Integrated mHealth App for Smoking Cessation in Black Smokers With HIV: Protocol for a Randomized Controlled Trial

**DOI:** 10.2196/52090

**Published:** 2024-04-24

**Authors:** Andre Bizier, Arielle Jones, Michael Businelle, Krista Kezbers, Bettina B Hoeppner, Thomas P Giordano, Jessica M Thai, Jacqueline Charles, Audrey Montgomery, Matthew W Gallagher, Marshall K Cheney, Michael Zvolensky, Lorra Garey

**Affiliations:** 1 University of Houston Houston, TX United States; 2 TSET Health Promotion Research Center Stephenson Cancer Center Oklahoma City, OK United States; 3 Department of Family and Preventive Medicine University of Oklahoma Health Sciences Center Oklahoma City, OK United States; 4 Massachusetts General Hospital Boston, MA United States; 5 Harvard Medical School Boston, MA United States; 6 Baylor College of Medicine Houston, TX United States; 7 HEALTH Institute Houston, TX United States; 8 Texas Institute for Measurement, Evaluation, and Statistics Houston, TX United States; 9 Department of Health and Exercise Science, University of Oklahoma Norman, OK United States; 10 The University of Texas MD Anderson Cancer Center Houston, TX United States

**Keywords:** smoking cessation, Black, HIV, anxiety sensitivity, mobile health, mHealth, just-in-time adaptive intervention, mobile phone

## Abstract

**Background:**

Black adults who smoke and have HIV experience immense stressors (eg, racial discrimination and HIV stigma) that impede smoking cessation success and perpetuate smoking-related health disparities. These stressors also place Black adults who smoke and have HIV at an increased risk of elevated interoceptive stress (eg, anxiety and uncomfortable bodily sensations) and smoking to manage symptoms. In turn, this population is more likely to smoke to manage interoceptive stress, which contributes to worse HIV-related outcomes in this group. However, no specialized treatment exists to address smoking cessation, interoceptive stress, and HIV management for Black smokers with HIV.

**Objective:**

This study aims to test a culturally adapted and novel mobile intervention that targets combustible cigarette smoking, HIV treatment engagement and adherence, and anxiety sensitivity (a proxy for difficulty and responsivity to interoceptive stress) among Black smokers with HIV (ie, Mobile Anxiety Sensitivity Program for Smoking and HIV [MASP+]). Various culturally tailored components of the app are being evaluated for their ability to help users quit smoking, manage physiological stress, and improve health care management.

**Methods:**

This study is a pilot randomized controlled trial in which Black combustible cigarette smokers with HIV (N=72) are being recruited and randomly assigned to use either (1) the National Cancer Institute’s QuitGuide app or (2) MASP+. Study procedures include a web-based prescreener; active intervention period for 6 weeks; smartphone-based assessments, including daily app-based ecological momentary assessments for 6 weeks (4 ecological momentary assessments each day); a video-based qualitative interview using Zoom Video Communications software at week 6 for participants in all study conditions; and smartphone-based follow-up assessments at 0, 1, 2 (quit date), 3, 4, 5, 6, and 28 weeks postbaseline (26 weeks postquitting date).

**Results:**

Primary outcomes include biochemically verified 7-day point prevalence of abstinence, HIV-related quality of life, use of antiretroviral therapy, and HIV care appointment adherence at 26 weeks postquitting date. Qualitative data are also being collected and assessed to obtain feedback that will guide further tailoring of app content and evaluation of efficacy.

**Conclusions:**

The results of this study will determine whether the MASP+ app serves as a successful aid for combustible cigarette smoking cessation, HIV treatment engagement, and physiological stress outcomes among Black people with HIV infection. If successful, this study will provide evidence for the efficacy of a new means of addressing major mental and physical health difficulties for this high-risk population. If the results are promising, the data from this study will be used to update and tailor the MASP+ app for testing in a fully powered randomized controlled trial that will evaluate its efficacy in real-world behavioral health and social service settings.

**Trial Registration:**

ClinicalTrials.gov NCT05709002; https://clinicaltrials.gov/study/NCT05709002

**International Registered Report Identifier (IRRID):**

PRR1-10.2196/52090

## Introduction

### Background

People with HIV/AIDS smoke at a rate 3 times higher (33.6% vs 12.5%) than that of the general population [1,2]. This group is also less likely to quit smoking and is more susceptible to the harmful effects of smoking (eg, increased risk for cancer and lung disease) [1,3,4], likely due to the combined impact of several social determinants of health, including behavioral risk factors, limited resources, and diminished access to health care, as well as immune system dysfunction and chronic inflammation caused by HIV [5-10]. Indeed, cigarette smoking is a leading risk factor for HIV-related and non–HIV-related morbidity and mortality among people with HIV/AIDS [11,12], even among those taking antiretroviral medications [13], and contributes to more life-years lost than HIV-related complications among those effectively treated with antiretroviral therapy (ART) [13-15]. Furthermore, people with HIV/AIDS who smoke are less adherent to ART than nonsmokers and experience poorer viral and immunologic responses to ART [16,17], a greater risk of virologic rebound, and more frequent immunologic failure [18].

Among people with HIV/AIDS who smoke, those who identify as members of a racial minority group are at an elevated risk for negative health consequences of smoking and poorer HIV-related outcomes. Evidence suggests that Black adults with HIV, in particular, experience high rates of discrimination and stigma that lead to the onset and maintenance of maladaptive coping, including smoking [19,20]. Consistent with these data, Black adults with HIV are more likely to smoke [4], less likely to quit [4,21-24], and experience greater quit difficulty when attempting to quit than White smokers or smokers without HIV [21]. Black adults with HIV also have lower rates of compliance with routine HIV care (ie, ART adherence), retention in long-term care, and are less likely to be virally suppressed relative to other groups [25-28]. This is a public health problem because Black adults account for the highest proportion (42%) of new HIV diagnoses and experience higher mortality than White adults with HIV [25,27,28]. From an intersectional stigma and discrimination perspective and socioecological models of social determinants of health [29,30], Black adults who smoke and have HIV may be at elevated risk for worse HIV disease management and smoking outcomes and ultimately experience increased health disparities [6].

Emotional models of coping suggest that Black adults with HIV may smoke and continue to smoke despite health problems to manage interoceptive stress and uncomfortable physiological arousal associated with minority status stressors such as racial discrimination and HIV-related stigma. Anxiety sensitivity (AS) is one of the most noteworthy constructs related to physiological distress and manifestations of elevated internal distress, including psychopathologies. AS refers to the fear of anxiety or anxiety-related symptoms [31]. AS amplifies negative mood states via enhanced threat perception (eg anxiety) [32,33], contributing to the development of anxiety and depressive problems [34]. Notably, anxiety and depression contribute to an increased likelihood of poor ART adherence [35]. Among people with HIV/AIDS, AS is related to more severe social anxiety symptoms, anxious arousal symptoms, HIV-related stigma, and HIV symptom distress [36,37]. AS has also been implicated as a contributing factor in smoking initiation, maintenance, and relapse [38,39]. Emerging data indicate that AS is elevated in both Black adults who smoke and people with HIV/AIDS who smoke [40], placing this group at greater odds of early lapse and relapse [41,42]. Without appropriate interventions to address susceptibility to the negative impact of interoceptive stress, Black adults with HIV who smoke and have elevated AS may be inclined to return to smoking to alleviate abstinence-induced increases in anxiety and to manage uncomfortable HIV-related bodily symptoms that may increase with smoking cessation.

Although smoking cessation treatments that target AS exist, they are limited in their reach, adaptability, and potential for adoption and do not consider critical barriers to smoking cessation or HIV treatment engagement and adherence. For example, current combined AS and smoking treatments focus primarily on multisession, intensive treatments, particularly those that rely on in-person, clinician-administered psychosocial protocols [43-46]. Participation in these treatments often requires notable time commitments, practical limitations, and expense [47]. Such burdens are barriers to treatment and may contribute to the low treatment participation rates [47]. An additional limitation of current treatments is that no readily available smoking cessation treatments that target AS integrate information to improve HIV treatment engagement and adherence. Given the robust evidence that targeting AS and promoting smoking cessation may lead to improved HIV outcomes, this is a major limitation. Finally, the required human resources, specialized training, and financial support that are needed to administer currently available combined AS and smoking treatments discourage the adoption of these treatments in communities most in need, such as those that serve Black adults with HIV who smoke. Therefore, it is essential to develop a digital AS intervention that can be culturally adapted for this population.

### Objectives

On the basis of our prior work [48], we modified our previously developed and tested novel, integrated, smartphone-delivered intervention for AS reduction and smoking (ie, Mobile Anxiety Sensitivity Program [MASP]) [49] for Black people with HIV/AIDS who smoke, integrating HIV information and care support that is culturally tailored to create the new smartphone-based app: Mobile Anxiety Sensitivity Program for Smoking and HIV (MASP+). MASP+ contains features and content designed to aid Black adults with combustible smoking cessation, HIV care adherence, and AS reduction. The initial efficacy of MASP+ relative to an established control intervention, the National Cancer Institute’s (NCI) QuitGuide app, on combustible cigarette smoking cessation, HIV-related outcomes, and AS reduction is currently being tested in a pilot randomized controlled trial (RCT). We expect that MASP+ participants will report greater biochemically confirmed smoking abstinence at 26 weeks postquitting date relative to the QuitGuide group. In addition, we hypothesize that MASP+ participants will report greater HIV-specific quality of life, ART adherence, and HIV treatment engagement at 26 weeks postquitting date relative to the QuitGuide group. Finally, we expect that improvement in AS will mediate the relationship between treatment and (1) smoking abstinence and (2) HIV-related outcomes and that daily experiences of discrimination (race and HIV) will moderate these relationships. We will also examine qualitative and quantitative data to guide the refinement and further adaptation of MASP+ and support the development of a high-quality, culturally relevant, and scalable intervention.

## Methods

### Ethical Consideration

The Institutional Review Board at the University of Houston (UH) approved the protocol presented in this study under STUDY00003811, whereas the University of Oklahoma Health Sciences Center and Baylor College of Medicine relied on the UH Institutional Review Board. This trial has been registered at ClinicalTrials.gov (NCT05709002; protocol ID: QKWEF8XLMTT3).

### Study Eligibility

The study eligibility criteria included being aged ≥18 years, having HIV, self-identification as Black or African American, providing a current picture of their cigarette package to verify smoking status [48], possessing at least a grade 6 reading level (≥4 on the Rapid Estimate of Adult Literacy in Medicine [REALM]) [50], experiencing elevated AS (ie, Short Scale Anxiety Sensitivity Index [SSASI] score of ≥5) [51], reporting daily smoking with a minimum of 10 cigarettes per day for at least 2 years, being motivated to quit smoking (>5 on a 10-point scale) [52], willingness to complete all study surveys or assessments, agreeing to use nicotine replacement medications (nicotine replacement therapy [NRT]; nicotine patch and lozenges), and agreeing to attempt to quit smoking 2 weeks after the date of randomization. Exclusion criteria included not being fluent in English; high blood pressure that is not under control (eg, medicated); experiencing a heart attack (myocardial infarction) within the past 2 weeks; use of any pharmacotherapy targeting smoking cessation beyond what is provided by this study; legal status that would interfere with participation (eg, incarceration with restricted access to mobile devices); cognitive impairment (assessed via the 6-item Cognitive Impairment Test) [53]; and being non-Black, pregnant, or younger than 18 years.

### Recruitment and Procedures

**Figure 1:**
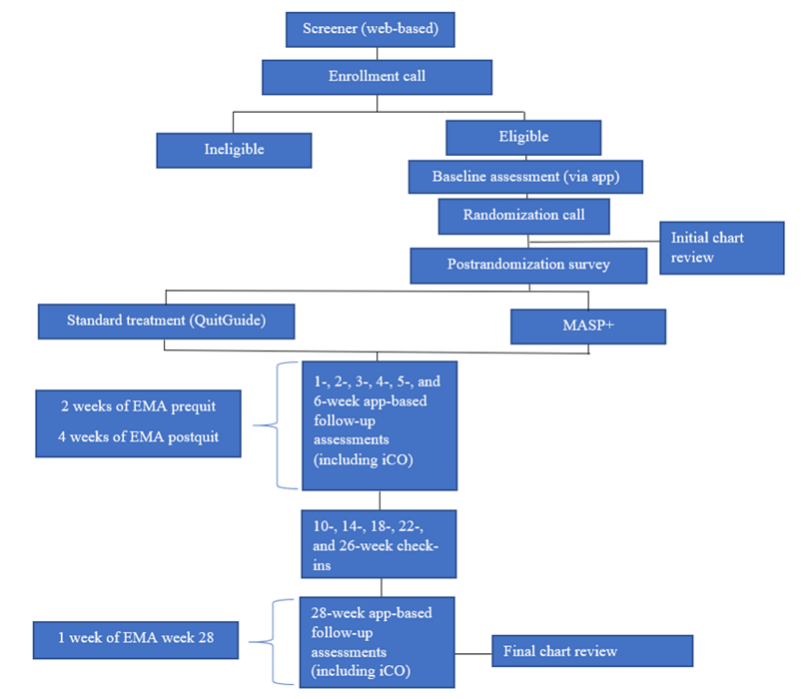
Mobile Anxiety Sensitivity Program for Smoking and HIV (MASP+) participant enrollment flowchart. Note: after initial eligibility screening, participants will complete an enrollment call, baseline survey, randomization call, postrandomization survey, 6-week ecological momentary assessment (EMA) period, monthly check-ins for 5 months, and a final survey and week of EMAs at week 28. iCO: iCarbon Monoxide.

Participants are recruited via web-based advertisement materials, as well as physical materials placed throughout the city of Houston. Study materials provide contact information for UH if a potential participant has questions about the study. Study advertisements also include a QR code linked to a REDCap (Research Electronic Data Capture; Vanderbilt University) prescreener survey. Those who complete the prescreening are deemed either ineligible or pre-eligible. For those recruited from Thomas Street at Quentin Mease Health Center, a clinic in Houston that provides routine medical care to ≥5000 people with HIV/AIDS study team members at Thomas Street perform a chart review to confirm HIV status. Pre-eligible participants are then contacted to complete an enrollment call wherein they provide informed consent and complete the final eligibility screening for the study (refer to Figure 1 for the participant enrollment flowchart).

Persons deemed eligible at the enrollment call download the Insight app onto their personal smartphone or study phone. The Insight app houses the MASP+ content and is used to administer smartphone-based surveys and ecological momentary assessments (EMAs) to all participants. The study smartphones are provided to those without a smartphone and those with a smartphone that is incompatible with the Insight platform. Insight is supported on most Android smartphones (Android versions 6.0-14.0). Participants with a personal phone that is incompatible with Insight are sent a study phone. For those using their personal smartphone, the study staff instruct participants on how to download the app onto their personal phone. Insight adjusts according to the font size selected by participants on their phones. Participants who experience issues reading text in the app due to font size are instructed to increase font size in the phone settings. Once Insight is downloaded, participants are given a unique single-use code that enables them to use the app to complete the baseline assessment, which must be completed within 6 days after inputting the code. If participants do not complete the baseline survey within 6 days, they are no longer eligible to participate in the study. All participants are mailed a baseline package that includes a Bedfont iCO (iCarbon Monoxide) quit smokerlyzer, a Greenphire Mastercard, and NRT. The Bedfont iCO is used to biochemically verify self-reported abstinence at follow-ups.

Upon completion of the baseline survey and receipt of the baseline package, the participants complete a randomization call with the study staff. During the randomization call, the participants are randomized to receive the MASP+ or QuitGuide interventions based on the randomization chart developed by the study statistician (MWG). Block randomization was used and stratified according to binary sex (assigned at birth). Variable-sized permuted block randomization (block sizes vary from 4 to 12) is used. Before data analyses, the balance of randomization will be checked and controlled for imbalanced factors by including any factors that differ between groups as covariates. The participants are provided with a unique code to access the app to which they are randomized. MASP+ participants have access to all MASP+ features (ie, Treatment Videos, Coping Toolkit, Quit Tips, and Stress Management Trainings) through the Insight platform. Participants randomized to the control intervention (ie, QuitGuide) have access to limited features (ie, App Instructions, Payment Tracking, Call Staff, and Record Stress) through the Insight platform and receive intervention content (ie, Track My Craving, Manage My Mood, and Learn To Quit) through the QuitGuide app. Participants assigned to the control intervention receive assistance with downloading the QuitGuide app through the Google Play Store. Both intervention groups use the Insight app to complete the study assessments, which include the baseline survey, weekly follow-ups, the 28-week follow-up, and EMAs. Once the code is entered, the research staff orients the participant to the app features and the iCO carbon monoxide (CO) breath testing device. Participants have access to MASP+ or QuitGuide content through the final follow-up (ie, the 28-week follow-up). At the end of the randomization call, participants are directed to take a brief postrandomization survey, which must be completed within 6 days after the randomization call. Failure to complete the postrandomization survey within 6 days does not affect participants’ status in the study.

Following randomization, the participants complete 4 daily EMAs for 6 weeks. Participants also complete app-based follow-up surveys each week of the treatment period (ie, weeks 1-6) and at 26 weeks postquitting date (28 weeks postbaseline). Between weeks 6 and 28, all participants are prompted to complete a brief check-in survey each month, which reminds them about available app features and assesses their current smoking status. The participants complete a final series of EMAs (4 per day) for 7 days before the 28-week follow-up assessment. The Insight app prompts daily EMAs and follow-up assessments for both treatment groups. In addition, participants complete a Zoom-based qualitative interview to assess their experience with the app and capture recommendations for improving app features and content at 6 weeks postrandomization. Participants who received loaned study smartphones are required to return them after their final follow-up assessment at week 28.

### Compensation

Each participant who enrolls receives a Greenphire Mastercard gift card that is loaded with compensation for survey completion. Participants receive all compensation in USD. Participants receive US $30 for completing the baseline assessment and US $10 for completing the postrandomization survey. Participants receive US $10 for completing each weekly follow-up assessment at weeks 1, 2 (quit date), 3, 4, and 5 as well as 6 weeks postbaseline (including the app-based survey, iCO breath test, and Zoom-based qualitative interview at week 6 only). Participants receive US $50 for completing the 28-week follow-up assessment (via Insight) and iCO breath test. Participants are compensated at the end of week 6 for EMA completion during weeks 1 to 6 and at the end of week 28 for EMAs completed during week 28. Specifically, participants receive a total of US $60 for completing 50% to 74% of the brief EMAs (4 per day×7 days=28 weekly EMAs) prompted during weeks 1 to 6, US $90 for completing 75% to 89% of EMAs, or US $120 for completing ≥90% of EMAs. For week 28, participants will receive US $10 for completing 50% to 74% of the brief EMAs, US $15 for completing 75% to 89% of EMAs, or US $20 for completing ≥90% of EMAs. Participants can use the “Payment” and “Weekly Survey Payment” buttons on the app’s home screen whenever desired to view an up-to-the-moment summary of EMAs presented and their current completion rate. Payments for completed EMAs are loaded onto participants’ Greenphire cards following week 6 and week 28.

### Study Conditions

#### Both Conditions

**Figure 2:**
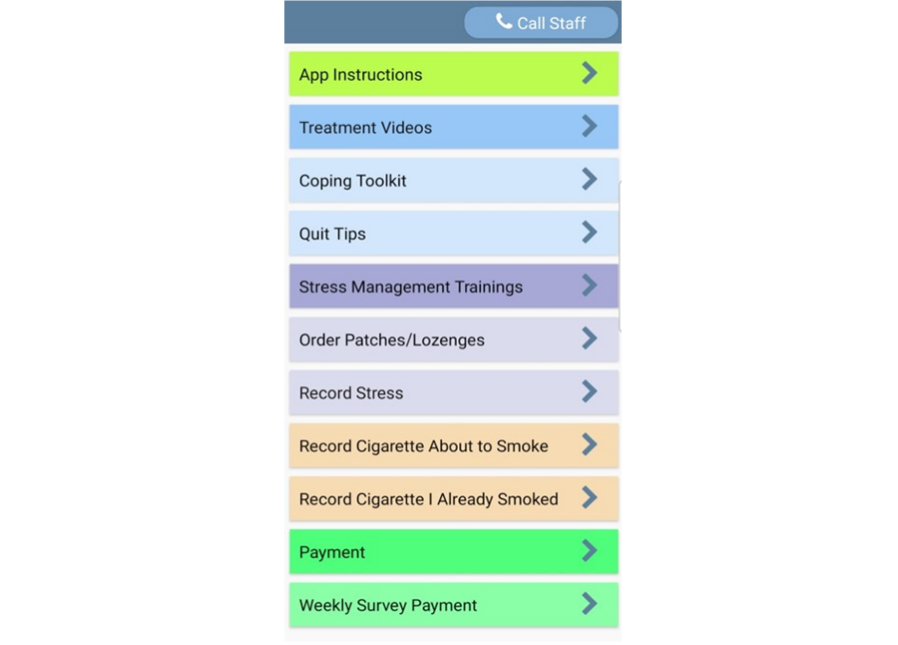
Mobile Anxiety Sensitivity Program for Smoking and HIV (MASP+) app home screen. Note: app features include App Instructions, Treatment Videos, Coping Toolkit, Quit Tips, Stress Management Trainings, Order Patches/Lozenges, Record Stress, Record Cigarette About to Smoke, Record Cigarette I Already Smoked, Payment, and Weekly Survey Payment.

Given that clinical guidelines recommend that all smokers attempting to quit should receive and use pharmacotherapy [54], both MASP+ and QuitGuide participants are sent NRT with their baseline package. Transdermal nicotine patches and nicotine lozenges are provided for use during the first 4 weeks postquitting date, and each participant is given the option order up to 4 weeks of additional NRT. For those in the MASP+ condition, an additional NRT can be ordered by clicking a button on the app home screen, which sends an encrypted email to the study team informing them of NRT requests. For those in the QuitGuide condition, NRT orders are placed by calling the number provided to participants and speaking with a member of the study team (Figure 2). In a prior study, 66% of enrolled participants used a similar app button or feature to place NRT orders [55]. Patches and lozenges were chosen for use in this study because of their safety and effectiveness (especially in combination with NRT), ease of use, and relatively benign side effects compared to other forms of NRT [56,57].

#### NCI QuitGuide Condition

The NCI’s QuitGuide app (available for free by request from the NCI Smokefree [58] server, on Google Play, or from the Google Play Store) is a free smartphone app that complies with many of the clinical practice guidelines for quitting smoking [54]. QuitGuide aims to orient smokers to their own smoking patterns and support users in developing the skills needed to quit smoking. Participants with a compatible smartphone who are randomly assigned to use QuitGuide are directed to download the app to their personal devices. Those who do not own a compatible device receive a study smartphone and are directed to download the QuitGuide app during the randomization call. Information explaining how to use the QuitGuide app is shared and reviewed during the randomization phone call. QuitGuide participants also download and use a modified version of the Insight EMA app with all MASP+ intervention features removed, which allows participants to receive EMAs and baseline or follow-up assessments. Refer to Table 1 for a list of QuitGuide features.

**Table 1:** Comparison of treatment conditions^a^.

App components			MASP+^b^		QuitGuide
EMA^c^			✓		✓ (add on for this study)
Set a quit date			Quit date is set to 2 weeks after randomization		Quit date is set to 2 weeks after randomization
Share quit information on social media					✓
Smoking cessation psychoeducation			✓		✓
Content specific to Black smokers with HIV			✓		
NRT^d^ tips and use advice			✓		
AS^e^ psychoeducation			✓		
**On-demand tips and exercises**					
	Coping with cravings	✓		✓	
	Coping with mood	✓		✓	
	Coping with stress	✓			
	Coping with HIV stigma	✓			
	HIV care management	✓			
	Inspirational messages	✓		✓	
Scheduled tips					✓
Treatment messages tailored to currently present smoking lapse triggers			✓		
**Coping toolkit**			✓		
	Guided relaxation and mindfulness exercises	✓			
	Challenging automatic thoughts	✓			
	Tips for coping with stress	✓			
	Interoceptive exposure	✓			
	Resources to help distract participants if they experience elevated distress	✓			
Individualized quit plan					✓
Document smoking triggers					✓
List reasons for quitting					✓
Savings from smoking fewer cigarettes					✓
Create journal entries					✓

^a^Some treatment components will be available to all participants regardless of group or app assignment during randomization (eg, smoking cessation psychoeducation and inspirational messages). Other components will be exclusive to either the MASP + condition (eg, content specific to Black smokers with HIV and guidance for challenging automatic thoughts) or the QuitGuide condition (eg, scheduled tips and individualized quit plan).

^b^MASP+: Mobile Anxiety Sensitivity Program for Smoking and HIV.

^c^EMA: ecological momentary assessment.

^d^NRT: nicotine replacement therapy.

^e^AS: anxiety sensitivity.

#### MASP+ Condition

The MASP+ app was adapted from MASP materials and focuses on smoking cessation, HIV treatment engagement or adherence, and AS reduction among Black smokers with HIV [59,60]. Specifically, treatment video scripts were updated from MASP to reflect language and life experiences particular to those with HIV (eg, “Smoking can cause infections, slow down healing, and make it harder for HIV medications to keep your immune system strong.”). EMA messages underwent similar tailoring (eg, “Thinking about the stress of HIV treatment can sometimes feel overwhelming. Relieve your stress by doing one of the relaxation exercises in the app’s Coping Toolkit.”), and 2 new features were added to the app: “How to Improve my Treatment Outcomes” and “Tips on Living with a Chronic Disease.”

Within a culturally adapted framework, MASP+ integrates both standard cognitive behavioral therapy practices for smoking cessation (in accordance with clinical practice guidelines) and transdiagnostic treatment for AS reduction [54]. MASP+ provides participants with (1) scheduled treatment content, (2) participant-initiated and scheduled stress exposure sessions, (3) personalized messages following each completed EMA (both pre- and postquit), and (4) numerous “on-demand” features (eg, Quit Tips and Coping Toolkit; both pre- and postquit). Culturally tailored components (eg, educational content related to menthol tobacco products in the Black community, the history of tobacco marketing directly to Black people, HIV treatment management and HIV disparities, and the impact of discrimination and racism on smoking and stress) are featured throughout MASP+. To support cultural tailoring, subject matter experts, including coinvestigators specializing in health disparities and HIV research and members of a Community Research Advisory Board, provided feedback on the cultural tailoring of the study materials for Black participants with HIV.

### MASP+ App Features

#### Treatment on a Schedule

MASP+ includes 17 videos that are 4 to 6 minutes long. These videos provide psychoeducation on topics such as nicotine withdrawal, unhelpful thinking, coping with others smoking nearby, managing uncomfortable sensations, chronic stress and HIV, myths about smoking, strategies for cessation and relapse prevention, smoking as a temporary coping mechanism to avoid experiencing negative emotions, thinking flexibly, stress management, stress and smoking, interoceptive exposure techniques, and the importance of using NRT. All videos were based on those deployed in the MASP study; however, scripts and content were updated to reflect the life experiences of Black adults with HIV (eg, smoking can cause infections, slow down healing, and make it harder for HIV medications to keep your immune system strong). In addition, 1 video was split into 2 videos to reduce the length of individual videos and thereby reduce participant burden. Two new videos become available each day for the first 8 days of the intervention. Participants are able to watch videos as they become available or later by clicking on the on-demand “Treatment Videos” button (Figure 2). There is no limit on how frequently each video can be viewed. The app records date-, time-, and location-stamped information each time a video is watched. This process occurs at both initiation and completion.

#### Exposure Sessions

Empirical studies demonstrate that internet-based stress exposure is well tolerated, acceptable, and effective [61,62]. The MASP+ treatment videos introduce graduated exposure to anxiety- and distress-provoking situations and response prevention to target AS. Originally, these exposure exercises were created for the MASP pilot study (that is, straw breathing, running in place, head rush, overbreathing, and chair spinning) [49]. Participants are instructed to press the “Stress Management Trainings” button to initiate a stress exposure session (Figure 2). The MASP+ app randomly selects 1 of the 5 exercises each time a participant presses the Stress Management Trainings button. The participants are then guided through stress exposures: the app explains to the participant the purpose of the assigned activity and how to perform it, normalizes the physiological symptoms experienced during the exercise, and relates this experience to their quit attempt. As in the MASP pilot [49], when the participant is ready to begin, the app assesses their level of distress (0-100 scale), displays a countdown timer while the exercise is being completed, and then assesses their level of distress again (0-100 scale) following the expiration of the countdown. The app suggests repeating the exercise up to 3 additional times if the participant’s current reported distress is >50 on a 1 to 100 scale. The aim of this strategy is to increase habituation to feared physiological sensations. Participants in the MASP pilot study accessed the Stress Management Training exercises on 6 out of 13 days before their quit date [63]. A recent review of phone-delivered interventions for anxiety and depression showed that treatments involving interoceptive exposure were safe and effective for participants [61,62,64].

#### EMAs With Tailored Real-Time Treatment Messages

During the prequit date period (ie, a participant’s first 2 weeks in the study), the MASP+ app delivers a message at the end of each EMA (4 per day) intended to increase motivation and provide information about quitting (eg, “Everyone experiences negative emotions, such as stress. These emotions do not last forever, but they can lead to relapse. Make a specific plan to cope with such feelings.”). During the postquitting date period (weeks 3-6 postbaseline), participants receive tailored messages following each EMA. These messages are based upon their responses to the EMA items they answered, which assess constructs such as ART adherence (eg, “Did you miss a dose of your HIV medication yesterday?”) and reported the likelihood of smoking today (ie, 0%-100%). HIV-related EMA messages were specifically developed for the MASP+ study. When participants report that they missed a dose of medication, they are prompted to select the reason that they missed their medication (eg, “Simply forgot” or “Away from home”). On the basis of the response or responses they provide, a tailored message is shared with the participant. For example, selecting that a dose was missed due to forgetting leads the app to display 1 of several possible messages to encourage participants to take their medication as prescribed (eg, “Taking your medications every day is a step toward improving your health!”). The type of message (eg, motivational, coping with urges or stress, and tips for HIV care management) that is delivered following each EMA is recorded and uploaded to our server for future analyses that examine the effects of messages on targeted smoking lapse triggers, HIV outcomes, and anxiety or depression in future EMAs. In addition, participants are instructed to review and practice stress management exercises (ie, interoceptive exposure exercises) to normalize and learn to manage symptoms of anxiety and withdrawal. These exposure exercises have been deployed without incident in the MASP study [49].

#### On-Demand Features

**Figure 3:**
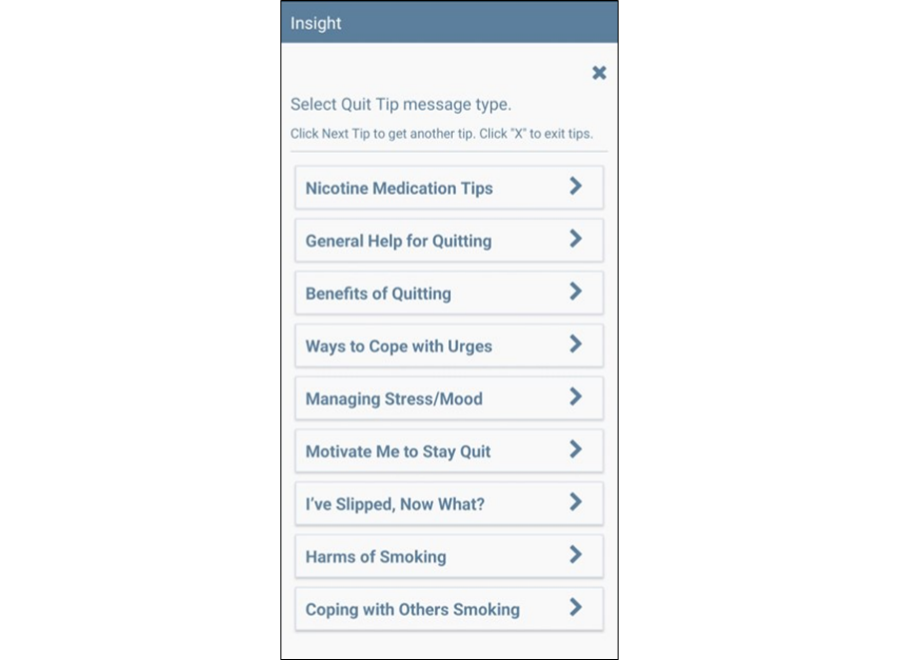
Mobile Anxiety Sensitivity Program for Smoking and HIV (MASP+) quit tips feature. Note: the Quit Tips button will bring participants to a screen where they can select to receive advice on the following topics: Nicotine Medication Tips; General Help for Quitting; Benefits of Quitting; Ways to Cope with Urges; Managing Stress/Mood; Motivate Me to Stay Quit; I’ve Slipped, Now What?; Harms of Smoking; and Coping with Others Smoking.

**Figure 4:**
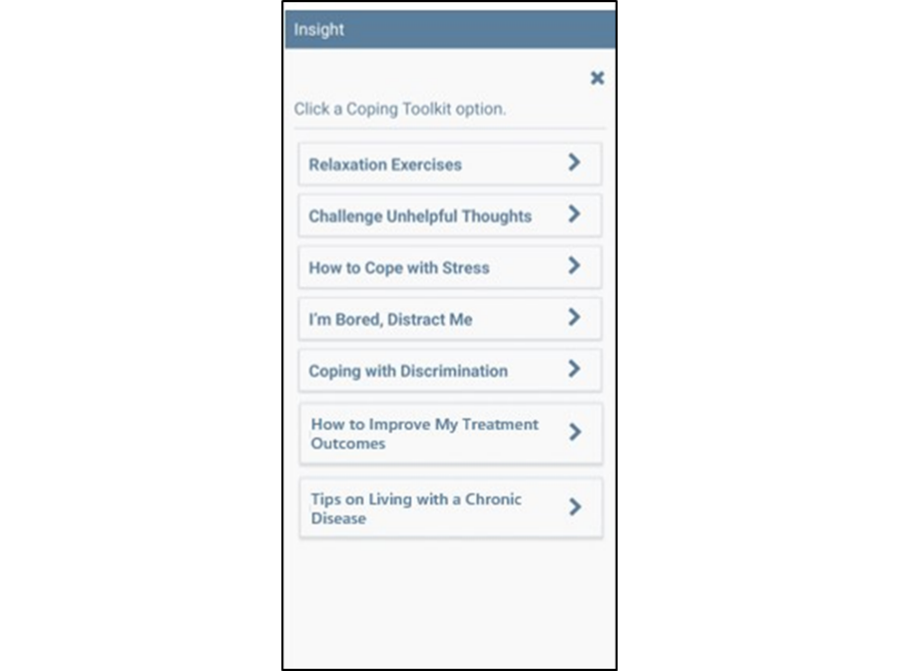
Mobile Anxiety Sensitivity Program for Smoking and HIV (MASP+) coping toolkit feature. Note: the Coping Toolkit button will bring participants to a screen where they can select to receive help with the following topics: Relaxation Exercises; Challenge Unhelpful Thoughts; How to Cope With Stress; I’m Bored, Distract Me; Coping With Discrimination; How to Improve My Treatment Outcomes; and Tips on Living With a Chronic Disease.

On-demand features including Quit Tips (Figure 3) and Coping Toolkit (Figure 4) are available to participants via buttons found on the MASP+ home screen. Each of the available icons provides a specific message related to the content area or an activity to support participants in that moment. The research team has developed hundreds of unique messages that address various triggers for smoking relapse [55,65]; the large message bank is intended to reduce repetition. Two new on-demand features were developed for this study: How to Improve My Treatment Outcomes and Tips on Living with a Chronic Disease. When selected, these features provide on-demand tips and messages of support regarding managing HIV care (eg, “Set reminders on your phone to help you remember appointments and when to take your medication.”) and coping with health stress (eg, “Going on a daily walk, even if just for a few minutes, can help you relax and sort through your thoughts!”), respectively. LG and MB led the development of these messages and activities.

### Measures

#### Overview

To reduce the potential for data entry errors and the need to retain paper copies of raw data, baseline and follow-up data are collected via smartphone using the Insight mHealth platform app software [66]. Questions appear on the phone screen, and answers are provided via touch screen. Tables 2-4 provides the schedule for when each measure is administered. The baseline survey takes approximately 30 minutes to complete; the postrandomization survey takes approximately 10 minutes to complete, the EMAs take approximately 2 to 4 minutes to complete, and the follow-up assessments take up to 15 minutes to complete.

**Table 2:** Table of measures^a^.

Survey measures	Total number of items, n	Screener, n	Baseline, n	Postrandomization, n	Week 1 to week 6, n	Week 28, n
Screener Survey	41	41	—^b^	—	—	—
6-Item Cognitive Impairment Test (cognitive screener; eligibility)	7	7	—	—	—	—
Rapid Estimate of Adult Literacy in Medicine-Short Form [50] (eligibility)	9	9	—	—	—	—
Demographics or background information	19	—	19	—	—	—
MacArthur Scale of Subjective Social Status	2	—	2	—	2	2
Everyday Discrimination Scale	10	—	10	—	—	—
HIV adherence and treatment	17	—	—	17	—	17
Qualitative interview	24 (MASP+^c^); 20 (QG^d^)	—	—	—	24 (MASP+ week 6); 20 (QG)	—
ART^e^ adherence	6	—	6	—	6 (week 6)	6
AIDS Clinical Trials Group Adherence Questionnaire	24	—	24	—	19 (week 6)	19
Index of Engagement in HIV Care	10	—	10	—	10 (week 6)	10
WHO^f^ Quality of Life-HIV	36	—	36	—	8 (week 6)	8
Carbon Monoxide Analysis (Phone Bedfont iCO^g^)	1	—	1	—	1	1
SSASI^h^ (eligibility)	5	5	5	—	5	5
System Usability Scale	10	—	—	—	10 (week 6)	—

^a^Various measures will be deployed during the eligibility screener, baseline and randomization surveys, weekly surveys, and daily EMAs. Daily EMAs will include items assessing sleep quality, HIV medication adherence, and discrimination, whereas random and event EMAs will include items assessing social interactions and smoking availability.

^b^Not available.

^c^MASP+: Mobile Anxiety Sensitivity Program for Smoking and HIV.

^d^QG: QuitGuide.

^e^ART: antiretroviral therapy.

^f^WHO: World Health Organization.

^g^iCO: iCarbon Monoxide.

^h^SSASI: Short Scale Anxiety Sensitivity Index.

**Table 3:** Daily diary ecological momentary assessment.

Survey measures	Items	Prequit morning	Prequit evening	Postquit morning	Postquit evening
Smoking behavior	3	1	1	3	2
Sleep quality	2	2	—^a^	2	—
Distress levels	2	2	—	2	—
HIV adherence	2	2	—	2	—
Other substance or medication use	4	2	2	2	2
General health or quality of life	2	—	2	—	2
Discrimination	2	—	2	—	2
Motivation levels	2	—	1	1	2
NRT^b^ use and side effects	8	—	—	8	—
Watch a brief video	1	1	1	—	—
Confidence levels	4	—	—	3	4
Single-item anxiety assessment	1	—	—	1	1
Single-item depression assessment	1	—	—	1	1
Location	1	—	—	1	1
Take lozenge now	1	—	—	1	1

^a^Not available.

^b^NRT: nicotine replacement therapy.

**Table 4:** Random and event sampling ecological momentary assessment.

Survey measures	Items	Prequit	Postquit
Smoking behavior and availability	3	3	3
Stress and coping	2	2	2
Social interactions	1	1	1
Motivation levels	1	1	1
Other substance or medication use	1	1	1
Confidence levels	3	3	3
Single-item anxiety assessment	1	1	1
Single-item depression assessment	1	1	1
Location	1	1	1
Coping toolkit	1	1	1
Take lozenge now	1	—^a^	1

^a^Not available.

#### Screening

The prescreener includes a demographic questionnaire that assesses demographics (eg, sex, age, race, and ethnicity), smoking history, motivation to quit, and smartphone and data plan details. The REALM-Short Form was used to assess literacy (>grade 6 English literacy level is required to complete EMAs) [50]. Participants read the REALM words from their phone screen back to the research assistant during the enrollment call. If a participant has trouble reading the words due to vision issues, they have the option to use their phone settings to enlarge the font. The 6-item Cognitive Impairment Test is used to assess impairment in cognitive function (participants with scores <8 will be excluded) [53]. After participants recruited from Thomas Street Clinic complete the prescreener, a team member at Thomas Street Clinic performs a chart review to verify HIV status and record relevant details of health history, including history of respiratory illnesses related to smoking (eg, chronic obstructive pulmonary disease; COPD), height or weight, and number of missed appointments in preceding months. All other participants progress without a chart review.

#### Smoking Outcomes

During each weekly follow-up survey, smoking status is biochemically confirmed using the Insight platform and the Bedfont Scientific iCO smokerlyzer device. Biochemically confirmed 7-day point prevalence abstinence (PPA) at 26 weeks postquitting date is the primary study outcome. This is consistent with best practices as well as most published smoking cessation RCTs (refer to the clinical practice guidelines) [54,67]. In their baseline package, participants receive the Bedfont iCO smokerlyzer, which they use to verify smoking status at each weekly follow-up assessment (weeks 1-6 and week 26 postquitting date). At the end of each assessment, the participants are directed by the Insight app to connect the iCO device to the smartphone (simply by pressing the power button on the device) and complete the iCO test. The date and time of completion are collected and saved to our server along with the test results. Our CO criteria are informed by numerous studies that have used CO cutoffs of <7 ppm [68-74]. The CO level is a validated indicator of smoking status and outcomes and is strongly correlated with cotinine and other biochemical measures that have longer detection windows [75-77].

#### HIV Outcomes

HIV outcomes are assessed using several measures. HIV-specific quality of life is assessed using the World Health Organization Quality of Life-HIV [78], a questionnaire comprising 36 items related to the quality of life in individuals with HIV. The items assess psychological well-being, functional limitations resulting from HIV status, pain, and the impact of HIV status on interpersonal relationships. ART adherence is assessed using 2 measures: the ART Adherence Scale [79], which consists of 7 items that assess adherence to ART medication regimens and attendance of HIV-specific medical appointments, and the AIDS Clinical Trial Group Adherence Baseline Questionnaire [80], which assesses ART adherence and reasons for missed medication, and nonadherence across 20 items. HIV treatment engagement is assessed using the Index of Engagement in HIV Care [81], a 10-item scale that asks participants about their experience of HIV care.

#### Discrimination

Race-based and HIV status–based discrimination is assessed using the Everyday Discrimination Scale [82]. This 5-item measure allows participants to report how often they face various forms of discrimination. Consistent with past work [83], the 5 items were adapted to assess both race-based and HIV status–based discrimination for a total of 10 items.

#### Anxiety Sensitivity

We assess AS using the SSASI [51]. The SSASI is derived from the ASI-3 and consists of 5 items that measure AS using a 5-point Likert-type scale (0=very little to 4=very much) [31].

#### MASP+ Feedback

We assess the perceived utility of MASP+ using the standardized System Usability Scale (a participant-completed, reliable, and valid metric for measuring usability and acceptability of technologies), which is administered during a 6-week follow-up survey [84-86]. Using a 5-point Likert-type scale (1=strongly agree to 5=strongly disagree), participants indicate their level of agreement with statements about the app’s usefulness (eg, “I thought the smoking cessation app was easy to use”).

#### MASP+ Feedback: Qualitative Interviews

Participant experience is assessed via a Zoom-based qualitative interview conducted at the end of treatment (week 6). The semistructured qualitative interview is conducted by trained members of the research team and audio recorded. These interviews focus on the ease of interacting with the app, the usefulness of app features, how the app can be improved, how sociocultural factors that affect Black smokers with HIV could be further woven into the intervention, and their willingness to refer the app to a friend. As participants in both study arms use the Insight app to complete EMAs and receive intervention content via the Insight app or QuitGuide app, the qualitative interview questions primarily concern participants’ experiences with Insight (MASP+ and EMA, or EMA only) and QuitGuide (intervention content). During the qualitative interviews, participants provide details about the features of the different apps that they did and did not like (eg, “What did you like about the app?”), how app features impacted relevant study outcomes (eg, “Which, if any, app features helped you manage your HIV better?”), and how the app or apps they used could be improved (eg, “How can we make the app fit better with your life experiences?”). Combined with the primary outcomes and other quantitative data, qualitative interview data will be used to refine MASP+, which will then be tested in a large-scale RCT.

#### Ecological Momentary Assessments

##### Overview

At present, EMAs are the most accurate way to measure phenomena in real-time, natural settings [87,88]. EMA items are less biased than traditional in-person assessments and identify fluctuations in key variables related to the study outcomes. EMA data are used to tailor the MASP+ treatment content and identify both treatment mechanisms and moments of high risk for smoking lapse.

The EMA methodology used in this study mimics that used in our previous studies and by other researchers [49,55,65,87,89-95]. The EMA items assess multiple constructs hypothesized to be related to HIV care and smoking lapse. Three types of EMAs are used in this study: daily diary, random sampling, and event sampling. Random and daily diary EMAs are prompted and initiated by the app. The phone audibly and visually notifies the participants of these EMAs for 30 seconds. If participants do not respond after 5 prompts, the assessment is recorded as “missed. All assessments are date and time stamped for future analyses.

##### Daily Diary

Each morning during weeks 1 to 6 and week 28, participants complete a daily diary EMA, for which they receive a notification 30 minutes after waking. Likewise, participants receive a notification to complete their evening daily diary 75 minutes before their reported bedtime. Questions from the morning daily diary ask about thoughts, experiences, feelings, and behaviors from both the previous day and the present (eg, “Did you miss a dose of your HIV medication yesterday?” and “Today, how long ago did you last smoke a cigarette?”). The participants are also asked about smoking cessation medication use, ART adherence, and sleep quality from the previous day. During evening daily diaries, participants are asked about thoughts, experiences, feelings, and behaviors from the same day (eg, “How is your health in general today?” and “How would you rate your quality of life today?”).

##### Random Sampling

Twice each day during weeks 1 to 6 and week 28, participants are prompted to complete EMAs that are scheduled to occur randomly during each participant’s normal waking hours. Participants rate their affect by indicating their level of agreement with several statements on a 5-point scale from “strongly disagree” to “strongly agree” (eg, “I feel stressed”). In addition, participants are asked about smoking triggers (eg, “I have an urge to smoke”), current depression level (“Rate your current level of depression [feeling sad]”), and anxiety level (“Rate your current level of anxiety [feeling nervous]”). Participants are also asked to describe their current environment (eg, home and work) and social setting.

##### Event Sampling

Throughout the treatment period, participants are asked to initiate Smoking Assessments (prequit date), Lapse Assessments (postquitting date), and Stress Assessments (pre- and postquitting date).

Before their quit date, participants are instructed to click a Record Cigarette button every time they feel they are about to smoke or have already smoked a cigarette. In addition, 10% of the time, this triggers a brief survey asking participants about their affect, stress, and experiences while smoking (eg, “Smoking improved my mood” and “Smoking was pleasurable”). These smoking assessments are date, time, and location stamped for future analyses.

Participants are instructed to press the Record Cigarette I Am About to Slip or the I Already Slipped button and complete a lapse assessment each time they smoke after their quitting date. Items on these assessments are similar to those presented in the random and smoking assessments and worded to separately assess the participants’ responses immediately before and after the lapse.

Participants are instructed to press the Report Stress button and complete the resulting assessment each time they “experience a significant increase in stress.”

Importantly, each MASP+ pre- and postquitting date EMA is followed by a treatment message that is tailored to the participant’s responses and current situation. Those assigned to the QuitGuide group complete EMAs identical to the MASP+ group, although they do not receive tailored intervention messages after completion.

##### EMA Alert Settings

During the enrollment call, the participants’ sleep-wake times for each day of the week are recorded and stored (Note: these sleep-wake times can be changed by calling the study team). This practice is intended to prevent or reduce the chance of the phone ringing while participants are asleep. When an EMA is prompted, participants can delay (“snooze”) EMAs by up to 30 minutes by clicking the Snooze Assessment option on the smartphone screen.

##### Data Loss Prevention

To avoid potential data loss as a result of participants losing their phones, each device is programmed to synchronize and upload encrypted data with our secure server multiple times each day (Note: in most studies, <1% of phones have been lost) [48]. These procedures prevent the collected EMA data from being lost, allow the researchers to remotely monitor participants’ EMA completion rates, and identify when participants need to be contacted for low survey completion. Notably, EMA data are encrypted and password protected on each study phone. Therefore, the study data are only accessible to the research team. The lost phones are wiped remotely. Participants who lose their phones are provided up to 1 replacement.

### The Insight Platform

#### Overview

The Insight mHealth platform was developed by the mHealth Shared Resource at the University of Oklahoma Health Sciences Center and the Stephenson Cancer Center. This platform offers resources to help researchers build, test, and launch technology-based assessment and intervention tools [66]. The mHealth resource uses a sizeable team that includes 1 program manager, 4 project coordinators, and 4.5 computer scientists or engineers. Together, they develop and maintain web and mobile apps and relational databases. These apps are developed using cutting-edge, cross-platform design tools.

#### Smartphone Training

Our team has developed and implemented a brief user-friendly training protocol to aid those with limited smartphone experience. During the randomization call, all participants, depending on condition assignment, receive training on how to use either the MASP+ app or the EMA-only Insight app and the QuitGuide app. Both versions of the Insight app (MASP+ and EMA only) contain an “App Instructions” button to remind participants of how each app feature functions (Figure 2). In previous studies, similar protocols have resulted in high EMA compliance rates (eg, 82%-87% of all EMAs completed) in samples of socioeconomically disadvantaged and nondisadvantaged adults [55,65,95,96]. All smartphones used in this study automatically collect data on intervention delivery (eg, number of times features are used and number of minutes treatment videos are watched).

### Data Analyses

#### Overview

To test the hypothesis that MASP+ produces higher rates of smoking abstinence compared to the NCI QuitGuide app, a biochemically verified measure of 7-day PPA that is collected during weekly assessments via Bedfont iCO will be used. PPA is defined as no smoking, not even a puff, in the 7 days before follow-up assessments and biochemical verification of smoking abstinence (ie, CO levels <7 ppm according to an iCO test). We will calculate odds ratio effect sizes (with 95% CI) to estimate between-group differences for PPA at each follow-up assessment. The effect size for the comparison between conditions at the 26-week postquitting date assessment will serve as the primary analysis of the impact of treatment condition on smoking cessation.

We will then conduct a series of conditional latent growth curve models (LGM) to examine the impact of treatment condition on abstinence trajectories. First, an LGM will be specified using CO breath tests at 2 (quit date), 3, 4, 5, 6, and 28 weeks. The intercept of these models will be centered on the baseline assessment and will be specified to model linear change across the major assessments. A dummy code representing the treatment condition will be specified as a predictor of the intercept and slope factors to quantify the effect of MASP+ on the longitudinal course of abstinence.

The impact of treatment conditions on HIV quality of life, ART adherence, and HIV treatment engagement (hypothesis 2) will be examined using the same sequence of analyses: between-condition effect sizes followed by conditional LGM. We will then conduct univariate LGM to explore changes in AS a function of treatment (hypothesis 3), followed by a series of parallel process LGM to examine how changes in AS relate to changes in smoking and HIV outcomes. The indirect effects of treatment via AS will be evaluated by calculating bootstrapped CIs of the indirect effect using the MODEL INDIRECT command in MPlus.

#### Mixed Methodology

Triangulation mixed methods quantitative and qualitative data analysis will be used to evaluate quantitative and qualitative data [97]. Using an explanatory sequential mixed methods design, qualitative data collection and analysis will increase the investigators’ understanding of participant experiences of the tailored material for both cultural relevance and relevance to living with HIV.

#### Quantitative Data

Quantitative data analysis will focus on (1) behavioral markers of engagement with the app (ie, completion of >75% of all assigned videos and completion of >75% of all assigned exercises); (2) self-report evaluations of the app including ease of interacting with the app (ie, >75% of all participants agreeing that MASP+ is easy to use based on a rating of ≥3 on the scale), that the MASP+ features (eg, automated treatment messages that follow EMA and treatment videos) are useful and helpful on a similar scale, and that at least 75% of participants would be likely to recommend the app to a friend; and (3) data from the System Usability Scale [98-100]. Low engagement and evaluation of MASP+ will be discussed with each participant during the qualitative interview.

#### Qualitative Data

Qualitative data analysis will focus on participants’ experiences with the app and incorporating their feedback to improve the app interface and features. Individual interviews will be transcribed following completion of participant treatment and then reviewed by the research team to monitor data quality. The transcribed interviews will be coded using NVivo (version 12; Lumivero). Following a first reading of the transcripts, the interviews will be coded using 2 coding passes [101]. The first coding pass will be use focused [102], with initial codes developed from the question path questions and additional codes based on responses to the System Usability Scale. The qualitative researcher (MKC) will code the interviews, and the coding will be reviewed by a second member of the research team trained in qualitative methods. The coding disagreements will then be discussed and resolved. Memos and notes will be reviewed and discussed. A second coding pass will then be conducted based on additional codes identified during the discussion, which may be process based or theoretically based, depending on what emerges from the first round of coding. Following the review of both coding passes, the research team will conduct a thematic analysis in several steps, beginning with the MASP+ participants, where themes will be identified both within and between codes. The team will then create data displays and review the patterns of responses between related sets of codes [103]. Points of integration between the MASP+ qualitative and quantitative data will be identified, a joint analysis will be conducted, and joint displays will be created [104]. Following the joint analysis, the transcripts will be read again for additional confirming and disconfirming evidence of themes [103], and representative quotes will then be selected. The QuitGuide responses will then be analyzed and compared to the MASP+ responses.

### Sample Size Determination

As this is the first empirical evaluation of the MASP+ intervention in this population, our focus is on determining the feasibility of the new intervention and obtaining preliminary estimates of effect sizes on smoking and HIV outcomes and the hypothesized mechanism of change, rather than conducting a full-scale and statistically powered examination of comparative efficacy. We expect that MASP+ will be superior to the NCI QuitGuide on all outcomes examined, but that the effects of MASP+ may vary and be larger for smoking outcomes than for HIV outcomes. On the basis of power calculations conducted using Repeated Measures and Sample Size and simulation studies identifying the sample sizes necessary to detect indirect effects [105,106], a target sample size of 72 would provide sufficient statistical power to detect a medium to large effect (Cohen *d*≥0.6) and an indirect effect for H3 if the treatment effect on AS is large (Cohen *d*=0.8) and the effect of AS on outcomes is medium to large (Cohen *d*=0.5). Our conclusions will primarily be based on effect sizes and associated CIs, and the effect size estimates (and associated CIs) from this trial will be used to guide future larger trials of comparative efficacy.

Qualitative interviews are conducted with all MASP+ and QuitGuide participants. The recommended minimum size for any subgroup is 20 interviews [107]. However, based on our prior work [48], interviews vary substantially in the content provided, so interviews will be conducted with all enrolled study participants in MASP+ and QuitGuide to meet the saturation of key study questions. Interviewing all MASP+ participants should be sufficient for the saturation of key study questions. The QuitGuide interviews will be used as a comparative group following the analysis of the MASP+ content, based on our prior work.

## Results

This study received IRB approval on November 29, 2022, under STUDY00003811 and began data collection on October 16, 2023. As of manuscript submission, a total of 9 participants have been fully enrolled in this trial. Data enrollment is scheduled to be completed by June 2024, and data collection is scheduled to be completed by December 2024. Data analysis will begin February 2025 and results will be published March 2024.

## Discussion

### Expected Findings

Data collection is currently underway. Results are expected to indicate that MASP+, relative to QuitGuide, will lead to improved biochemically verified 7-day PPA, increased adherence to ART, and improved attendance at HIV-related health appointments at 26 weeks postquitting date. Qualitative interviews will be integrated with quantitative data to further refine and adapt MASP+ and support the development of an iterative intervention that is high quality, culturally relevant, scalable, and ready for rigorous testing as part of an R01-level grant.

### Study Implications

This study is the first to culturally tailor a smoking cessation smartphone-delivered intervention that is integrated with AS reduction skills for Black people with HIV/AIDS who smoke and elevated AS. Smartphone interventions such as MASP+ have the potential to provide low-cost, scalable treatments to diverse populations who may not be as likely to have access to in-person treatment or may not have health insurance to cover costs. A 2021 survey found that 85% of US adults reported owning a smartphone [108], and other studies have found that smartphone ownership is high among minoritized populations (83% among Black adults) as well as individuals with low socioeconomic status (76% among those earning <US $30,000/y) [108]. Given the widespread ownership of smartphones, MASP+ has the potential to overcome traditional barriers to care and enhance the accessibility and reach of culturally appropriate, clinical-grade care for hard-to-reach populations.

Clinically, the cultural tailoring and integration of AS reduction skills within MASP+ represents a groundbreaking approach. Experiences of AS and related mental health factors (eg, anxiety, depression, and drug withdrawal or craving) can vary over time and among individuals [109]. By assessing these factors throughout the day and providing support as these symptoms arise, MASP+ constitutes a just-in-time tailored intervention for smoking cessation, AS, and HIV management. Such an approach may serve as a potential solution to address the documented health disparities experienced by Black people with HIV/AIDS who smoke [6,110]. MASP+ is also distinguished by the specificity with which it can respond to the unique stressors, needs, and challenges experienced by Black smokers with HIV and AS. This underscores the potential for MASP+ to serve as a stand-alone or adjunctive treatment for smoking cessation. Indeed, MASP+ offers personalized care to address factors that impede successful behavioral change in a culturally appropriate and person-centered manner, which may have strong implications for mitigating health disparities for Black smokers with HIV.

Understanding the role of AS in the process of quitting cigarettes and better managing HIV among Black adults with HIV has several theoretical implications. First, this work bridges together disparate yet complementary work on AS, smoking, and HIV [111,112]. Thus, findings from this trial may provide a holistic understanding of how these constructs relate to and influence one another. This greatly advances the current models for the role of interoceptive distress as a risk factor for worse smoking cessation and HIV-related outcomes [113-116]. Second, several models related to minority stress indicate the need for culturally tailored interventions, particularly those targeting smoking cessation [117-119]. Expanding these efforts to produce tailored interventions for Black smokers with HIV that focus on a mechanism implicated in worse health outcomes among this group (ie, AS) allows providers and researchers to address documented health disparities among this population in a culturally specific manner [120]. Further studies of the mechanisms underlying quit success among Black smokers with HIV and AS will guide future efforts to tailor smoking cessation interventions for this population. This study will provide insights about which risk factors to target during smoking cessation treatment among this group, the ideal timing of intervention efforts, and the preferred content of intervention messages.

### Anticipated Limitations

This study has several limitations. Daily EMAs can be disruptive as they are prompted throughout the day rather than clustered in a single study appointment. This could potentially discourage participation in the daily EMA component of this study. In addition, some study measures rely on self-reporting, and participants may not respond in real time. In addition, recruiting a population as specific as Black people with HIV/AIDS who smoke only combustible cigarettes may prove challenging for the study team. Finally, although participants will be responding to items while navigating daily life, thereby increasing ecological validity, this comes at the expense of depriving experimenters of a high degree of control over participants’ environmental conditions.

### Conclusions

This study may provide insights into precision medicine treatment that is not otherwise available by providing a smoking cessation and HIV management intervention with tailored treatment content based on the psychological and environmental context in real time. Pending tests of its efficacy, an intervention that is automated, scalable, and culturally informed could be easily incorporated into other real-world settings and aid in the reduction of health disparities. This novel mobile intervention has the potential to address the mental and physical barriers to smoking cessation and treatment engagement that are unique to Black people with HIV/AIDS. Despite any potential difficulties in recruiting such a specific group or capturing EMA data, the benefits of this study far outweigh any drawbacks.

Additional work is essential for the successful translation and cultural adaption of effective in-person smoking cessation interventions into mobile, remotely delivered treatments such as MASP+. Compared to current treatment options, mobile interventions have the potential to produce even greater cessation outcomes and provide wider access for historically oppressed and underserved populations such as Black smokers with HIV and high AS. In light of prior studies that demonstrate the feasibility of mobile smoking cessation technology [49,55,121], mobile treatments that integrate AS constitute a vital “next step” for addressing tobacco-related health disparities among Black smokers with HIV. This study will extend our work in this area as well as in the wider field of smoking–emotional disorder comorbidity by adapting and testing a fully automated, culturally tailored, mobile AS smoking cessation intervention for Black smokers with HIV. Future work will focus on testing the MASP+ app in a larger, fully powered efficacy trial; national dissemination of intervention materials; and implementation across diverse health care settings.

## Abbreviations

ART: antiretroviral therapy
AS: anxiety sensitivity
CO: carbon monoxide
EMA: ecological momentary assessment
MASP: Mobile Anxiety Sensitivity Program
MASP+: Mobile Anxiety Sensitivity Program for Smoking and HIV
NCI: National Cancer Institute
NRT: nicotine replacement therapy
PPA: point prevalence abstinence
RCT: randomized controlled trial
REALM: Rapid Estimate of Adult Literacy in Medicine
REDCap: Research Electronic Data Capture
SSASI: short scale anxiety sensitivity index
UH: University of Houston

## Data Availability

The data sets generated or analyzed during this study are available from the corresponding author upon reasonable request.
